# Outbreak of non-tuberculous mycobacteria in a paediatric bone marrow transplant unit associated with water contamination of needle-free connectors and literature review

**DOI:** 10.1038/s41409-021-01239-4

**Published:** 2021-06-23

**Authors:** Stephanie Habermann, Elena Ferran, James Hatcher, Hermione Lyall, Josu De La Fuente, Elizabeth Whittaker

**Affiliations:** 1 Imperial College NHS Healthcare Trust, London, UK; 2 Great Ormond Street Hospital, London, UK

**Keywords:** Paediatrics, Preventive medicine

## To the Editor:

Non-tuberculous mycobacteria (NTM) are ubiquitous acid-fast bacteria existing in soil and water. They are divided into rapidly growing mycobacteria (RGM), which generates colonies on solid culture media within 7 days of incubation and slowly growing species which take weeks to grow. NTM lung disease is the most common clinical presentation of NTM disease. However, the clinical presentation of NTM systemic infections is often nonspecific and include fever, weight loss and lymphadenopathy. Disseminated disease may also lead to respiratory, gastroenterological or neurological presentations. Immuno-compromised patients such as oncology patients, children with primary immune-deficiencies and those undergoing solid organ and haematopoietic stem cell transplant (HSCT) [1] are at higher risk of developing systemic NTM infections . The incidence of NTM infections is rare but in HSCT patients it is between 50 and 600 times higher than in the general population [2]. A number of risk factors for NTM infections in patients undergoing HSCT have been identified, these include type of graft, conditioning regimen, persistent immunosuppression and prolonged presence of central venous catheter (CVC) [1].

Following HSCT, the most common presentation is central line associated bloodstream infection (CLABSI) (36%) [2]. Treatment of CVC-related NTM infections can pose problems due to formation of a biofilm on the surface of the CVC which is difficult to eradicate. Prognosis for HSCT patients with CVC-related NTM infections is generally good [1].

CLABSI caused by NTM is often caused by water colonisation. A recent systematic review of waterborne NTM hospital outbreaks described 21 distinct outbreaks [3]. All patients affected were susceptible due to a variety of immunodeficiencies or indwelling CVC. Of the 21 outbreaks, 5 occurred in haematology–oncology units and 4 in outpatient clinics. All patients in the haematology–oncology unit outbreaks were immunosuppressed and presented with CLABSI. Only one outbreak occurred on a paediatric unit but did not involve HSCT patients [3]. Here we describe the first outbreak of NTM in a paediatric bone marrow transplant (BMT) unit in England with water contamination of CVC and needle-free connectors (NC) implicated amongst other factors.

This outbreak investigation occurred on the paediatric haematology BMT unit at St Marys Hospital, a teaching hospital in London, between February and September 2010. The unit carries out approximately 35 allogeneic transplants yearly, mostly in children with haemoglobinopathies or red cell disorders.

A case was defined as any paediatric haematology patient with a positive blood culture (BC) for NTM in the 8-month period of the outbreak. Medical records were reviewed to obtain clinical and laboratory data including patient demographics, BMT type, conditioning regimen, presence of CVC and NC, clinical symptoms, positive BC, treatment and outcome. The absence of such cases during the same period the year before, led us to define it as an outbreak, especially as neither the surveillance policy nor the laboratory and blood sampling techniques changed.

In order to identify the potential source of infection, water samples were obtained from the paediatric BMT unit. The infection control practices of the department were reviewed, with a special emphasis on handling of CVC sites and their NC, as well as the relevant information provided to patients at discharge.

The patient characteristics of the five patients that developed NTM infections are described in Table 1. All of the patients had CVC with NC which were removed for source control. Three patients required subsequent replacement. All patients were treated with prolonged courses of antibiotics; amendment due to drug adverse events was required for all. No infection recurrence occurred, and all patients made a full recovery.

**Table 1:** Patient information.

Patient	1	2	3	4	5
Sex	M	M	M	F	F
Age (years)	4	3	7	16	13
Primary diagnosis	Beta thalassaemia major	Beta thalassaemia major	Fanconi	Sickle cell	Sickle cell
Reason for transplant	Regular blood transfusion from age 6 m, then chelation therapy. Liver biopsy: mild fibrous expansion of portal tracts and mild lymphocytic infiltrate	Regular blood transfusion from age 5 m, then chelation therapy. Liver biopsy: mild generalised fibrous expansions of portal tracts with mild portal and lobular inflammation	No complications yet	Frequent vaso-occlusive crisis requiring hospital admissions despite hydroxycarbamide and blood transfusions, avascular necrosis hip	Frequent vaso-occlusive crisis, acute ischaemic stroke resulting in left sided hemiparesis,
Conditioning	Busulfan 14 mg/kg, Cyclophosphamide 200 mg/kg, alemtuzumab 0.3 mg/kg	Treosulfan 42 g/m^2^, Cyclophosphamide 200 mg/kg, alemtuzumab 0.3 mg/kg,	Fludarabine 150 mg/m^2^, Cyclophosphamide 40 mg/kg, anti-thymocyte globulin 11.25 mg/kg	Busulfan 14 mg/kg, Cyclophosphamide 200 mg/kg, Alemtuzumab 0.3 mg/kg	Busulfan 14 mg/kg, Cyclophosphamide 200 mg/kg, Alemtuzumab 0.3 mg/kg
GvHD prophylaxis	Ciclosporin 1.5 mg/kg bd, Methotrexate 10 mg/m2 Day +4 and +7	Ciclosporin 1.5 mg/kg bd, mycophenolate mofetil 600 mg/m^2^ bd	Ciclosporin 1.5 mg/kg bd	Ciclosporin 1.5 mg/kg bd, Methotrexate 10 mg/m^2^ Day +4 and +7	Ciclosporin 1.5 mg/kg bd, Methotrexate 10 mg/m^2^ Day +4 and +7
Transplant type	Sibling	Maternal HLA matched BMT	Sibling	Sibling	Sibling
Day post BMT with NTM bacteraemia	−5	115	89	77	117
Days post line insertion with NTM bacteraemia	9	167	101	91	159
Presentation	Fever	Fever	Fever, rigors	Weight loss, nausea, vomiting and rigors	Fever
Neutrophil count	Normal	Neutropenia	Normal	Normal	Neutropenia
GVHD	No	No	No	No	Unknown
Isolate species	*Mycobacterium mucogenicum*	*Mycobacterium chelonae*	*Mycobacterium fortuitum*	*Mycobacterium chelonae*	*Mycobacterium spp- undefined*
Initial antibiotic therapy	Azithromycin, Amikacin, rifabutin	Clarithromycin, Amikacin and Meropenem	Clarithromycin, amikacin and meropenem	Clarithromycin, Amikacin, Meropenem, Rifabutin	Exact regime unknown—included clarithromyin
Change in antibiotic and reason	Rifabutin was stopped due to pancytopenia, 2 m later ceftriaxone added for consolidation	Meropenem stopped as resistant, clarithromycin stopped late in therapy due to high LFT	Rifabutin added.	Meropenem stopped, ciprofloxacin added, amikacin stopped due to nausea and vomiting, replaced with ethambutol	Clarithromycin stopped due high LFT
Duration (months)	3	6	3	3	Unknown
Co-infection	None	CoNS in BC and from line tip	None	Enterobacter cloacae (urine)	CoNS in BC
Hospital/community acquired	Hospital	Community	Community	Community	Community

*CoNS* coagulase negative staphylococcus, *LFT* liver function test, *BC* Blood culture.

All patients grew RGM from CVC BCs. The median time from CVC insertion to positive BC was 101 days (range 9–167). The median time from the stem cell infusion to positive BC was 89 days (range −5–117).

Atypical mycobacteria were identified in water samples from the BMT unit. This was discussed with Public Health England who felt that the significance of NTM in water samples was unclear. Subsequently, line care was identified as the main contributing factor to the outbreak. Further qualitative work, including guideline review and patient interviews, identified that patients showered and bathed at home without adequately protecting the CVC, a recognised source of contamination. Therefore, guidelines were modified, and health-workers and patients received further training on handling of CVC and NC. Sterile transparent semi-permeable dressings were provided for patient use when bathing and showering. Finally, the model of NCs was changed. No further NTM infections were detected following these interventions.

We report a cluster of RGM CLABSI in a paediatric BMT unit over an 8-month period. There were multiple factors contributing to this outbreak, and CVC and NC exposure to contaminated tap water was implicated. Updating guidelines and educating patients and health professionals were interventions that contributed to no further cases being identified.

A literature review identified eight previous outbreaks of CLABSI due to RGM [4–11]. A total of 43 patients were affected; 21 were children. All of these outbreaks occurred on haematology–oncology units and half involved HSCT patients [4, 7, 8, 11].

In this outbreak we identified similar RGM to previous outbreaks. However in previous outbreaks most patients were affected by the same organism whereas this outbreak was polymicrobial. A potential explanation is that in this outbreak most patients were outpatients and may have acquired the NTM from tap water at home. Despite this observation, we describe this as an outbreak as all patients were actively managed at one centre.

The presumed mechanism of contamination of the CVC and NC was with tap water during bathing and showering, when the lines were not adequately covered. Therefore, CVC handling guidelines were reviewed and NCs changed to a different model, which have been successful strategies in previous similar reports [4, 5, 8–11]. An education programme on CVC care for families was updated and education for healthcare staff refreshed.

In view of the uncertain significance of the water sampling results, polymicrobial results indicating multiple water sources and interventions being successful at preventing further outbreaks, no further action was required to address water source contamination.

In this cluster, all patients underwent CVC removal and treatment with prolonged courses of antibiotics, as recommended in general guidelines for management of mycobacterial CVC-related bacteraemia (14). However, as there are currently no specific guidelines for the treatment of NTM infections in HSCT recipients, treatment is quite variable. All previous outbreaks had CVC removed but only half were managed with prolonged courses of antibiotics [5, 7, 9, 10]. Previous reports support short courses of antibiotics, and given the frequent side effects associated with prolonged antimicrobial treatments, shortening treatment of NTM bacteraemia in patients without disseminated disease should be considered. Further research into optimal length of treatment and development of NTM treatment guidelines outside of pulmonary disease would be a step forward in the management of NTM in immuno-compromised patients.

In conclusion, this report highlights that NTM should be considered as a cause of fever in immuno-compromised patients with CVC especially as morbidity is high and treatment long and costly. Successful strategies for disease prevention included: increasing awareness of potential sources of infection, including water and NC and ensuring regular patient and staff education regarding management of CVC and NC.
